# *De Novo* Assembly of the *Streptomyces* sp. Strain Mg1 Genome Using PacBio Single-Molecule Sequencing

**DOI:** 10.1128/genomeA.00535-13

**Published:** 2013-08-01

**Authors:** B. Christopher Hoefler, Kranti Konganti, Paul D. Straight

**Affiliations:** Department of Biochemistry and Biophysics, Texas A&M University, College Station, Texas, USAa; Whole Systems Genomics Initiative, Texas A&M University, College Station, Texas, USAb

## Abstract

We report a draft genome assembly of *Streptomyces* sp. strain Mg1, a competitive soil isolate with multiple secondary metabolite gene clusters.

## GENOME ANNOUNCEMENT

*Streptomyces* sp. strain Mg1 is a competitive soil bacterium with a complex secondary metabolism. An intriguing characteristic of Mg1 is its ability to cause lysis and degradation of *Bacillus subtilis* cells and colonies (1). The genome was sequenced to enable prediction of secondary metabolites and determination of their relative contributions to the competitive functions of Mg1.

Whole-genome shotgun sequencing of the Mg1 strain was carried out using PacBio SMRT sequencing technology (2). For assembly of the Mg1 genome, we applied the recently described hierarchical genome assembly process (HGAP) to eight SMRT cells of sequencing data generated from an 8- to 10-kb insert library (3). The final assembly is 8.7 Mb in seven contigs. Greater than 90% of the predicted genome size is contained within one large 7.8-Mb contig. The remaining sequence is divided into smaller contigs 50 to 500 kb in length.

The microbial genomes of *Streptomyces* sp. are challenging to sequence. These genomes are characteristically high in GC content and possess large (>8-kb) rRNA gene clusters (4, 5). Many contain biosynthetic gene clusters encoding polyketide synthases (PKSs) and nonribosomal peptide synthetases (NRPSs), large multimodular enzymes with repetitive domain structures (6). These sequence features are difficult to assemble using second-generation approaches, primarily because short read lengths limit the ability of assembly algorithms to resolve low-complexity and repetitive regions (7). Due to their extraordinary length, single-molecule sequencing reads are capable of spanning long repeats which, along with a distinct lack of coverage bias, greatly simplify the process of genome assembly. Previously, the Mg1 genome was assembled into 466 contigs (GenBank, ABJF00000000). Comparison to the current assembly shows many collapsed repeats that could not be resolved and nearly 1 Mb of sequence missing due to large coverage gaps. High coverage of short reads from 454 GS-FLX titanium and Illumina HiSeq 100-bp paired-end data did not resolve the problems with the previous assembly.

With this updated assembly, we were able to predict multiple secondary metabolite gene clusters using antiSMASH (8). All of the predicted PKS gene clusters from the previous assembly were broken at contig boundaries, including the one that produces chalcomycin A (9). In the current assembly, the chalcomycin gene cluster is fully contiguous along with seven other predicted PKS and NRPS gene clusters. Additional PKS and NRPS gene clusters remain fragmented, but the ability to mine the Mg1 genome for secondary metabolites and predict their structures has been greatly improved.

The maturation of single-molecule sequencing provides an unprecedented way for researchers to assemble and finish microbial genomes. Assembly of a nearly finished Mg1 genome using only PacBio technology illustrates the accessibility of single-molecule sequencing for studying the medically and industrially significant *Actinobacteria*. Improvements in genome contiguity and accuracy aid analyses that require large-scale ordering of the genome sequence, such as the mining of secondary metabolite gene clusters.

### Nucleotide sequence accession numbers. 

This whole-genome shotgun project has been deposited at DDBJ/EMBL/GenBank under the accession no. ATCJ00000000. The version described in this paper is the first version, ATCJ01000000.
